# Radiomic features analysis in computed tomography images of lung nodule classification

**DOI:** 10.1371/journal.pone.0192002

**Published:** 2018-02-05

**Authors:** Chia-Hung Chen, Chih-Kun Chang, Chih-Yen Tu, Wei-Chih Liao, Bing-Ru Wu, Kuei-Ting Chou, Yu-Rou Chiou, Shih-Neng Yang, Geoffrey Zhang, Tzung-Chi Huang

**Affiliations:** 1 Division of Pulmonary and Critical Care Medicine, Department of Internal Medicine, China Medical University Hospital, Taichung, Taiwan; 2 Department of Medical Imaging, Chang Bing Show Chwan Memorial Hospital, Changhua, Taiwan; 3 Department of Biomedical Imaging and Radiological Science, China Medical University, Taichung, Taiwan; 4 Artificial Intelligence Center for Medical Diagnosis, China Medical University Hospital, Taichung, Taiwan; 5 Department of Radiation Oncology, Moffitt Cancer Center, 12902 USF Magnolia Drive, Tampa, FL, United States of America; 6 Department of Bioinformatics and Medical Engineering, Asia University, Taichung, Taiwan; Taipei Medical University, TAIWAN

## Abstract

**Purpose:**

Radiomics, which extract large amount of quantification image features from diagnostic medical images had been widely used for prognostication, treatment response prediction and cancer detection. The treatment options for lung nodules depend on their diagnosis, benign or malignant. Conventionally, lung nodule diagnosis is based on invasive biopsy. Recently, radiomics features, a non-invasive method based on clinical images, have shown high potential in lesion classification, treatment outcome prediction.

**Methods:**

Lung nodule classification using radiomics based on Computed Tomography (CT) image data was investigated and a 4-feature signature was introduced for lung nodule classification. Retrospectively, 72 patients with 75 pulmonary nodules were collected. Radiomics feature extraction was performed on non-enhanced CT images with contours which were delineated by an experienced radiation oncologist.

**Result:**

Among the 750 image features in each case, 76 features were found to have significant differences between benign and malignant lesions. A radiomics signature was composed of the best 4 features which included Laws_LSL_min, Laws_SLL_energy, Laws_SSL_skewness and Laws_EEL_uniformity. The accuracy using the signature in benign or malignant classification was 84% with the sensitivity of 92.85% and the specificity of 72.73%.

**Conclusion:**

The classification signature based on radiomics features demonstrated very good accuracy and high potential in clinical application.

## Introduction

Lung cancer remains the leading cause (Her2-neu and EGFR) was associated with inferior survival worldwide for both men and women [1, 2]. Early diagnosis of pulmonary nodules can improve the clinical outcomes. Early and accurate diagnosis plays an important role in the treatment of cancer [3]. Malignant lung nodules are related to extremely high mortality. The survival rate of benign lung nodules raise dramatically. Therefore, the accuracy and reproducibility of diagnosis for discriminating between benign and malignant nodules is essential.

Much of research on personalized medicine has focused on the molecular level which identifies the genomic or proteomic signatures. However, it is well known that most tumors are spatially heterogeneous which possibly represents a limitation in these techniques [4, 5]. As these methods are costly and time consuming, they are not easy to implement into clinical routine. Currently, invasive biopsy may help to determine the status of tumors. However, biopsies need to be taken from the lesion, which is a highly invasive procedure. Also, since tumors are spatially heterogeneous, only extract a small portion of the lesion may not accurately represent a complete characterization of the tumor. Moreover, medical imaging is one of the major and important technologies in clinical oncology for greatly improving the diagnosis and guidance [6, 7]. It is a noninvasive and widely used method during routine clinical practice. Indeed, Computed tomography (CT) is the most common imaging modality for detecting and diagnosing pulmonary nodules [8–10]. CT images of lung nodules show the difference of intensity with background and can be well detected due to strong contrast. Nevertheless, the conventional 1- or 2-dimensional measurement of tumor size sometimes may give rise to false positive results and the overdiagnosis can lead to unnecessary treatments [11–15]. Also, the characteristics of tumor on the CT images are generally described subjectively. Thus, it is necessary that the quantitative imaging biomarker can potentially be used as more comprehensive predictor and help to precisely estimate the probability of malignancy for detected nodules.

Radiomics refers to the comprehensive quantification of tumor phenotypes by applying large amounts of features from medical images [16, 17]. It extracts the high dimensional information from medical images using advanced feature analysis algorithms. This means that such mineable image texture, shape, and intensity features can be further applied to build a predictive model, which relates specific features to the tumor phenotypes. Additionally, this approach can reduce subjective variability and improve diagnostic efficiency compared to current qualitative evaluation strategies. In a previous study, Aerts *et*. *al*. reported several radiomics features which could be used to identify a general prognostic phenotype existing in different cancers and had clinical power [4]. Kim *et al*. also appraised several 1- or 2-dimensional measurements with discussion on their limits and introduced the potential imaging biomarkers with emphasis on the current understanding of their clinical usefulness [15]. In those studies, it is demonstrated that images are minable data, and the phenotypic differences of tumors can be correlated to radiographic findings. More specifically, the lesion-specific imaging features can capture about tumor phenotypes and may potentially have clinical significance to cancer detection. However, how to select the features from a large number of imaging data and make it robust is a challenge in the development of predictive model.

In this study, the radiomics analysis is applied as a quantitative method to detect pulmonary nodules, and evaluate the relevance of specific features to phenotypes.

## Materials & methods

**Fig 1:**
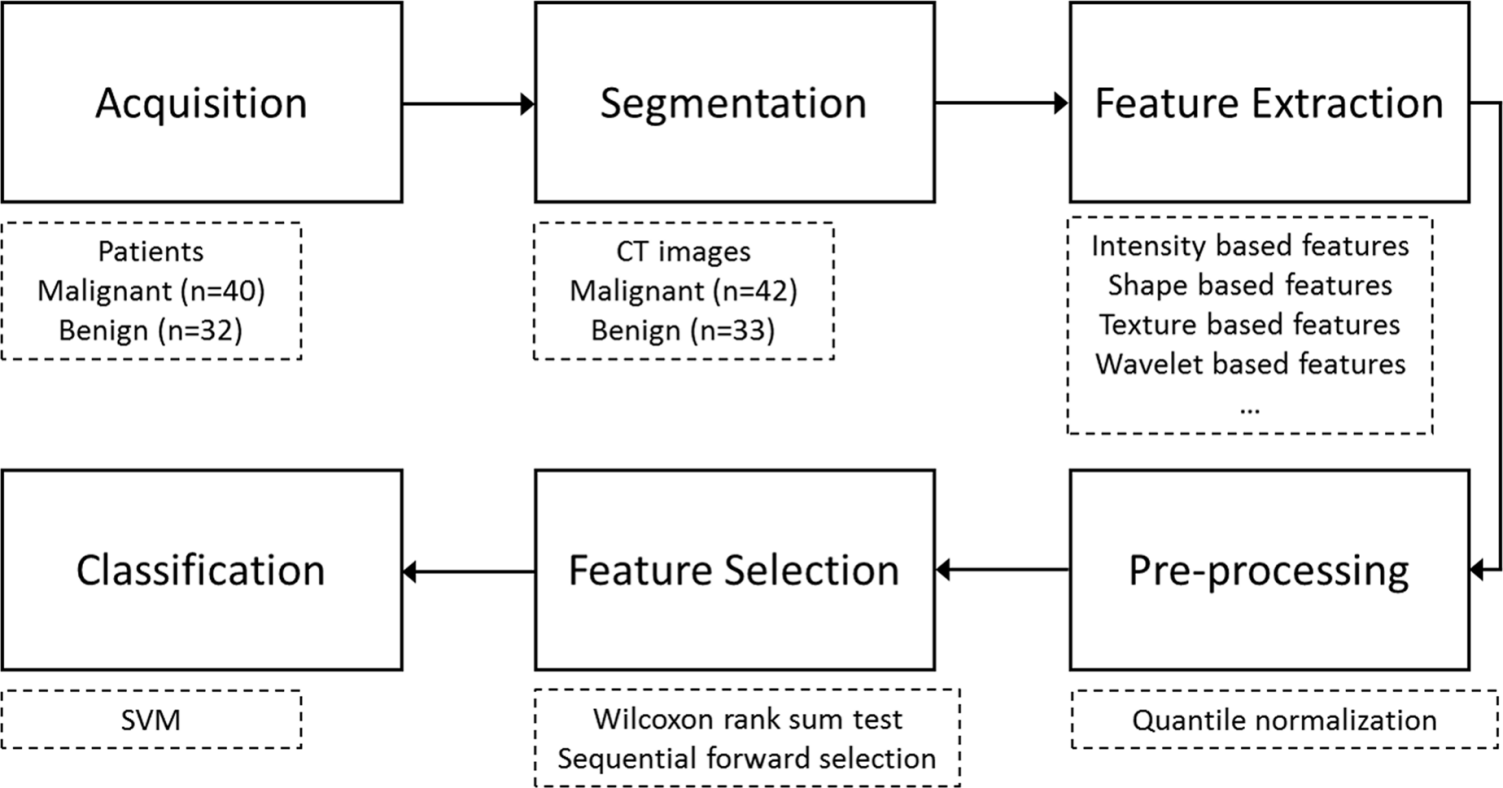
Radiomics analysis workflow. First, the clinical CT images of malignant and benign pulmonary nodules were collected. Second, image segmentation was used to delineate the pulmonary nodules. Next, the image features were extracted by the automated high-throughput feature analysis algorithm. Finally, the statistical analysis was applied and the sequential forward search was used for feature selection for the classification of lung nodules.

The overall workflow is shown in Fig 1. First, in the image acquisition step, the clinical CT images of malignant and benign pulmonary nodules from 72 patients were collected. Second, image segmentation was used to delineate the regions of pulmonary nodules. Next, the image features were extracted by the automated high-throughput feature analysis algorithm which provided a comprehensive characterization of lung nodules. Finally, the statistical analysis was applied as a filtering process and the sequential forward search was used for feature selection to build a multi-feature signature which should be high efficient and accurate in the classification of lung nodules. To summarize, the aim of this study was to find accurate image features that can be used in quantitative, precise and non-invasive medical diagnosis for lung nodules treatment.

### Patients characteristics

Standards of practice procedures at our institution were followed, and a written informed consent was obtained from each participant and the analysis performed in this study was approved by the Institutional Review Board of our institution (CMUH105-REC2-085). This study retrospectively collected the CT images of 32 (22 men and 10 women; age range, 14–82; mean, 55 years) and 40 (19 men and 21 women; age range, 37–85; mean, 59 years) patients with benign and malignant pulmonary nodules, respectively. The clinical pathological data including benign and malignant pulmonary nodule with cancer staging (TNM staging) are list in Table 1. As shown in the table, more than 6 kinds of benign nodules were found in this cohort of patients and the malignant nodules were distributed into 3 categories. A few patients had more than one nodule detected in the CT images. Thus the total number of nodules analyzed was 75 (42 malignant and 33 benign). The authors had access to information that could identify individual participants during data collection only.

**Table 1:** Characteristics of population.

**Histologically benign**	
Granulomatous inflammation	11
Organizing pneumonia	7
Cryptococcosis	4
Tuberculosis	3
Sclerosing hemangioma	2
Others	5
**Histologically malignant**	
Adenocarcinoma	36
Squamous cell carcinoma	3
Large cell carcinoma	1
**Tumor characteristics**	
Stage IA	23
Stage IB	6
Stage IIA	1
Stage IIB	3
Stage III	4
Stage IV	1
Unknown	2
**T-category**	
T1a	18
T1b	6
T2a	7
T2b	0
T3	6
T4	1
Unknown	2
**N stage**	
N0	34
N1	1
N2	3
N3	0
Unknown	2

### CT image acquisition

Non-contrast-enhancement CT images were taken under free-breathing condition for all the patients. A multi-detector row CT (Optima CT660, GE Medical System) was used for the acquisition of those CT images, with 120 kVp and smart mA. The image slice matrix was 512×512, with slice thickness being 5 mm and the pixel spacing 5×5 mm^2^, which were the default setting of the CT scanner for this anatomy site.

### Lesion delineation and segmentation

**Fig 2:**
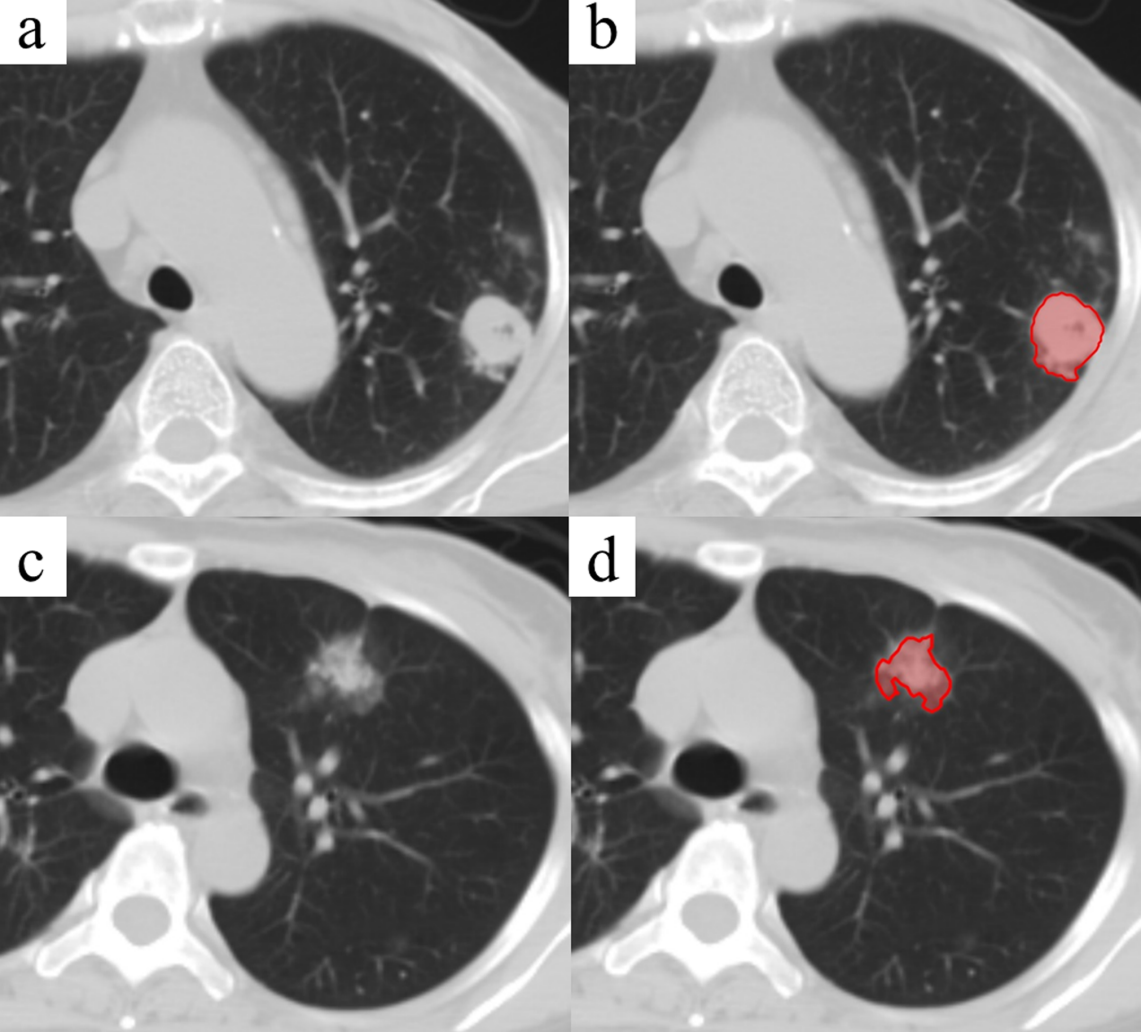
Examples of lung lesion segmentation. Original CT image (a) and target segmentation (b) of a benign lung lesion (tuberculosis) in patient’s left upper lobe. Another original CT image (c) and target segmentation (d) of a malignant lung tumor (adenocarcinoma) in patient’s left upper lobe.

All the CT images were loaded into the Eclipse radiotherapy treatment planning system (version 11.0, Varian Medical Systems, Palo Alto, California, USA). A radiation oncologist with twelve years clinical experience delineated the lesion volume which was used for the later feature extraction. No specific restriction was applied to the nodule selection. Any size, shape and category (solid, partial-solid, ground glass opacity or GGO) were included. Examples of lung lesion volume delineation were shown in Fig 2.

### Feature extraction

A total of 750 radiomics features per case were extracted from the expert-delineated lesions on non-contrast-enhanced CT images in this study. All feature calculations were performed by using a in-house software implemented on the Microsoft Visual Studio 2010 platform. Feature values are usually calculated using 3 different methods, statistical, modeling and transforming. The statistical based method uses the intensity distribution in the image to get the regional characteristics and derive various parameters [18].

First-order features are related to the characteristics of intensity distribution in the region of interest (ROI). They are usually based on the analysis of the intensity histogram. Such features include mean, maximum and minimum intensity values, standard deviation, skewness, kurtosis, uniformity and entropy [19]. Extended from the intensity histogram, Intensity-volume histogram (IVH) simplifies the complicated 3D information into a simple and easy to understand 2D curve. This concept is similar to the dose-volume histogram (DVH). For example, from the IVH, the intensity that covers a certain volume in the ROI can be determined. I30 is the intensity value that 30% of the ROI volume is covered by this value and higher; V40 represents the volume that is covered by the intensity value of 40% and up of the maximum intensity [20].

Second-order features include Co-occurrence Matrix (GLCM) features, proposed by Haralick et al [21, 22], and further developed and applied in applications by Liang [23]. The features are extracted based on the 2D matrix derived from the intensity relationship of the neighboring voxels in the 3D image. The intensities in the ROI of the 3D image are binned into a certain number of gray levels, i.e. 256. A 2D co-occurrence matrix with dimensions of 256×256 is then derived. The intensity relationship is checked in 13 directions in the 3D image set. The final matrix is an average result over the 13 directions. The matrix is converted into a probability matrix. The features are then calculated based on the 2D co-occurrence probability matrix. Run Length Matrix (RLM) is an L×R 2D matrix, where L is the number of gray levels (256 in this study) and R is the possible runs which is case dependent. A run is a group of voxels that have the same gray level in a certain direction. RLM is calculated in 13 directions, summed for all the directions and normalized. The second order features include entropy, uniformity, contrast, homogeneity, dissimilarity and correlation, etc. [18, 24].

Some other intensity based features are local regional characteristics related to the intensity differences between the neighboring voxels, which include coarseness (similar to granularity), contrast (represents the dynamic regional intensity variation and the variation range), business (the intensity variation rate) and complexity (sum of the normalized intensity variation) [25].

In this study, the statistical based texture features were extracted from lung lesion CT images. The feature categories include intensity and shape, Laplacian of Gaussian (LoG), wavelet, Laws, GLCM, RLM, Gray Level Size Zone Matrix (GLSZM), neighborhood gray-tone difference matrix (NGDM), fractal dimension. A description of radiomics features and associated feature category are listed in Table 2.

**Table 2:** Radiomics feature characteristics.

Radiomics feature	**# of features**
Intensity & shape based features	33
LoG based features	96
Wavelet based features	128
Laws features	432
Co-occurrence features	26
Run-length based features	11
GLSZM based features	11
NGTDM based features	5
Fractal Dimension features	8

Abbreviations: LoG = Laplacian of Gaussian, GLSZM = gray-level size zone matrix, NGTDM = Neighborhood Gray-Tone Difference Matrix.

### Statistics analysis of radiomics feature

To reduce the batch effects, in the feature analysis, the quantitative raiodmics raw data were normalized across all patients by using the Quantile normalization. It is important to select features, from the large number of features, that have discrimination power between benign and malignant lesions [26]. By using nonparametric Wilcoxon rank sum test, the significantly different features (p<0.05) between these two groups of cases were selected. The sequential forward selection (SFS) was then applied to further evaluate the correlation between the features and the groups. Support vector machine (SVM) was used as the classifier and the leave-one-out cross-validation method was applied to get the prediction accuracy for each feature. The most accurate one was selected followed by the next most accurate feature. Repeated the process until the number of features reached the desired number. Inclusion of more than 4 features did not improve the performance of the classifier. Therefore in this study, only top 4 features were selected to be the signature to identify different groups. All applied algorithms were implemented on Matlab R 2013a platform.

## Results

### Radiomics features selection

From the 750 extracted features, only some features from the 4 categories including intensity and shape, wavelet, Laws and run-length were found showing significant differences between the benign and malignant lesions (p<0.05). The numbers of features that showed such differences were 5 in intensity & shape, 4 in wavelet, 65 in Laws and 2 in run-length respectively (Table 3).

**Table 3:** Radiomics feature list that had significant difference (p<0.05) between malignant and benign groups.

Category of feature	Filter associated	Feature name	#
Intensity based features			5
	N/A	minI, maxI, meanI, Kurtosis, I30	
Wavelet based features			4
	LLH	min	
	LHH	min	
	HLH	contrast	
	HHL	lcl homo	
Laws features			65
	EEL	uniformity	
	EES	max, SD, RMS, energy	
	ELL	Kurtosis, energy, entropy	
	ELS	max, mean, SD, RMS	
	ESE	max, mean, SD, Coeff Vari, RMS, contrast, lcl homo	
	ESL	max, SD, RMS	
	ESS	max, SD, RMS	
	LEL	Kurtosis	
	LES	max, SD, RMS, energy	
	LLE	Peak, mean, Kurtosis	
	LLL	Kurtosis, energy, uniformity	
	LLS	min	
	LSE	max, SD, RMS	
	LSL	min, SD, Skewness, energy	
	LSS	max, SD, Coeff Vari, RMS, energy	
	SES	max, SD, RMS	
	SLL	energy	
	SLS	SD, RMS	
	SSE	max, SD, RMS	
	SSE	max, SD, RMS	
	SSL	Skewness, CV, energy	
	SSS	max, SD, Skewness, RMS	
Run-length based features			2
	N/A	LGRE, SRLGE	

Abbreviations: L = local convolution kernels; E = edge convolution kernel; S = spot convolution kernel; SD = standard deviation; RMS = root mean square error; CV = coefficient of variation

### Radiomics signature building

**Fig 3:**
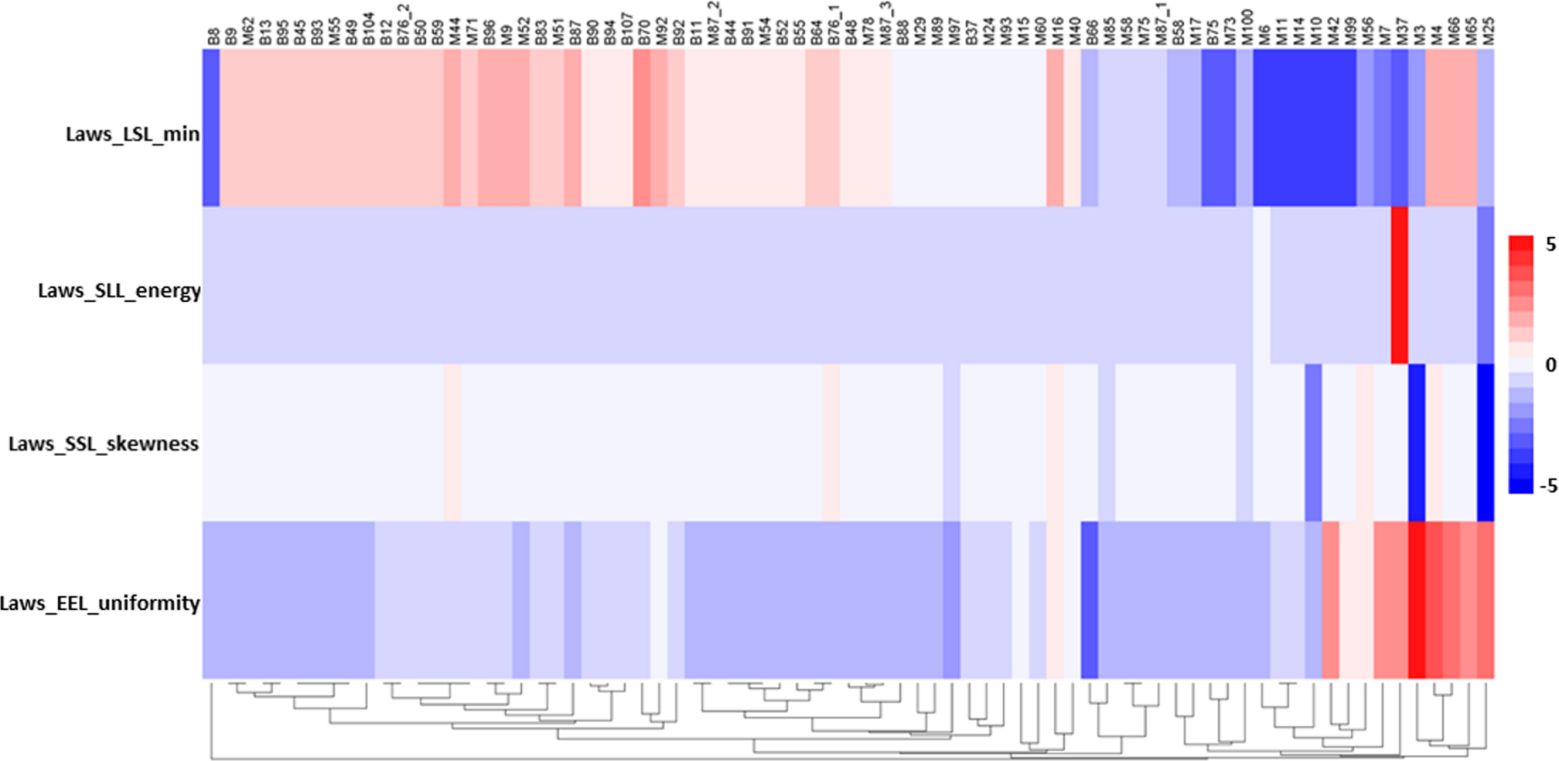
Heat map of the selected 4-features radiomics signature. Radiomics features expression with Z-score. Hierarchical clustering of lung lesions is on the x axis (n = 75, B = Benign, M = Malignant). The 4-feature radiomics signature expression is on the y axis.

The signature was built using the best 4 features from all the categories. These top 4 features were Laws_LSL_min, Laws_SLL_energy, Laws_SSL_skewness, Laws_EEL_uniformity. The 4-feature radiomics signature’s performance on all the pulmonary nodules was shown in Fig 3.

### Diagnostic performance of radiomics signature

**Fig 4:**
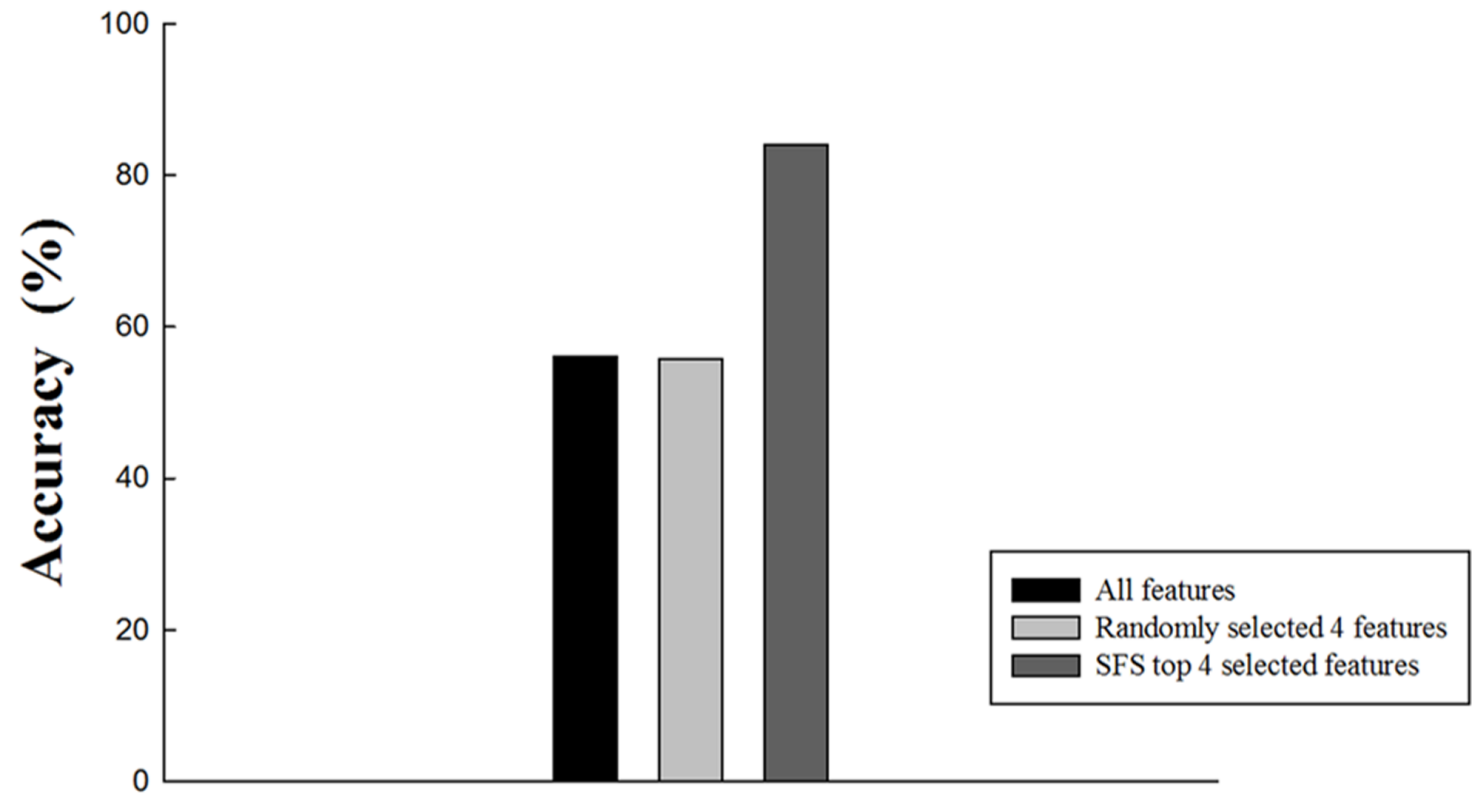
Prediction performance of the three different feature sets. A leave-one-out cross-validation was performed and the accuracies in the malignant and benign nodules were plotted. The randomly selected 4 features group was examined in a 1000-time permutation test.

Besides using the built 4-feature radiomics signature, signatures built by all features that showed significant differences between the two groups (76 features in total) and a random 4-feature from the 76 features were also applied to classify benign and malignant lesions as references. The accuracy was 56% when all features were used as the predictor, which demonstrated no prediction power. With a random 4-feature signature, 1000-time permutation test was performed and the mean accuracy was 55.8%. The accuracy for the selected 4-feature signature was 84% (Fig 4). Furthermore, with the 4-feature radiomics signature performance on various cases, the sensitivity was 92.85% and the specificity was 72.73% (Table 4), which demonstrated good prediction power.

**Table 4:** Selected feature.

Radiomic feature	Accuracy	Sensitivity	Specificity
The 4-feature signature (Laws_LSL_min, Laws_SLL_energy, Laws_SSL_skewness, Laws_EEL_uniformity)	84%	92.85%	72.73%

Abbreviations: L = local convolution kernels; E = edge convolution kernel; S = spot convolution kernel

## Discussion

In this study, radiomics method was applied to extract large number of radiomics features from pulmonary nodule patients’ CT images. After quantile normalization over the quantitative features, SFS was used over the normalized data to select and build 4-feature signature. The prediction power to classify benign and malignant pulmonary nodule of the 4-feature signature was then compared with that of all features and randomly selected signature.

Traditionally, invasive biopsy is often used in pulmonary nodule diagnosis to distinguish benign and malignant lesion. This method only exam partial lesion and the result cannot reflect the overall nodule which may cause errors in diagnosis [4, 5]. In addition, this invasive examination may introduce additional risk to the patient. For small nodules, follow-up to observe the development of the nodule is often the practical method used clinically. Further clinical action depends on the volume change of the nodule [27, 28]. The radiomics signature method introduced in this study is only applied on the routine clinical CT images. The patient does not take any additional surgery risk. And it does not have any limit on the nodule size and therefore enhances the possibility of proper treatment.

Some studies suggested that besides the biopsy exam to get knowledge regarding the histological characteristics, benign or malignant could be judged based on the nodule size. However, this often is not accurate [29]. A few recent studies evaluated the accuracies of physician direct diagnosis and diagnosis using established models and found that the accuracy by models was usually higher [30, 31]. This implies that the model or expression signature established through clinical data and validated algorithms can enhance the potential of better clinical diagnosis. Even more recent studies have attempted to improve the accuracy through the use of radiomics [17], and radiomics on pulmonary nodules [32, 33, 34] have improved accuracy of the pulmonary nodule classification. Based on the results in our study and previous radiomics results, the radiomics features extracted from the routine clinical CT images have the potential to be an non-invasive diagnosis method with high accuracy.

In a few studies, radiomics signatures were built by selecting features from different feature categories [4,32]. However, in this study, we tried to build signature with sequential forward selection method. It could be another way to generate signature with high accuracy of classification. The final features used in the radiomics signature were all from the Laws features in this study. This category of features mainly exams the target heterogeneity in the ROI, including the region microstructure, specifically spot, edge and level surfaces [33]. This is an implication that the heterogeneity inside a pulmonary nodule can be used to distinguish if it’s benign or malignant. The relationship between these features and the biological aspects inside the nodules needs to be studied in more details.

The benign lesions in this study covered quite different categories, including granulomatous inflammation, organizing pneumonia, cryptococcosis, tuberculosis, sclerosing hemangioma etc., as listed in Table 1. It should be easier to classify if in a single group the differences are small between cases. If in a group, there are different categories of lesions, the variation in the group would be large. However, even with this unfavorable condition, the 4-feature radiomics signature built in the study still had good capability in classification. For the benign group, the specificity was greater than 70% even with so many different lesions in the group. On the other hand, the greater than 90% sensitivity indicates that the signature is very sensitive in malignant lesion classification, which is clinically important. If a malignant lesion was diagnosed as a benign one, the treatment could be delayed which may cause serious problems.

In many other studies, the lesions size which is relative big or small were usually excluded [34]. However, in this study, there is no specific size restriction applied in case selection. The lesions size in our sample is from 10 mm to 90 mm in diameter. In addition, various texture types of lesions were included, including solid nodule, partial-solid nodule, GGO, etc. Even with such differences in size and texture, the 4-feature radiomics signature still showed excellent power in classification.

The values of many features are voxel size dependent [35]. In this study, all the CT images were acquired with the same resolution, thus the feature analysis should be valid. In the future, multi-center collaboration is in our plan to involve more cases in order to enhance the statistical power. The image resolution is likely to be different between centers. When image data from multiple centers are analyzed, resampling of the image data to a consistent resolution could be the way to go to avoid the features’ voxel size dependency issue.

In the future, the analysis workflow may be improved, which in turn may enhance the classification power. Many studies compared the manual ROI delineation and semi-auto segmentation and found that the semi-auto segmentation gave more consistent results [31]. Therefore applying semi-auto segmentation method instead of manual delineation in the study may reduce the inter-observer variation and enhance the efficiency. In addition, with more cases in the future, an independent dataset may be used as an external validation group for the radiomics signature, which may further evaluate the signature’s accuracy in classification.

A more significant limitation of this study is that the validation of the 4-feature radiomics signature was using same data set which is not independent may result in a bit overfitting in the result. In the future, for evaluation, the validation would run on the independent test data set and the performance obtained on the set would be more reflective of the real predictive power of current method.

## Conclusion

The radiomics signature that was composed of the best 4 radiomics features demonstrated high accuracy in lung nodule classification. The signature introduced in this study may provide a non-invasive method in clinical lung nodule classification. This method would allow early classification of lung nodules with comprehensive characterization.

## Supporting information

S1 File CT image data of lung nodule. (ZIP)
