# Supplementary figures and images for: Radiomic features analysis in computed tomography images of lung nodule classification
Source: PLoS One. 2018 Feb 5;13(2):e0192002. doi: 10.1371/journal.pone.0192002 (PMC5798832; doi:10.1371/journal.pone.0192002)

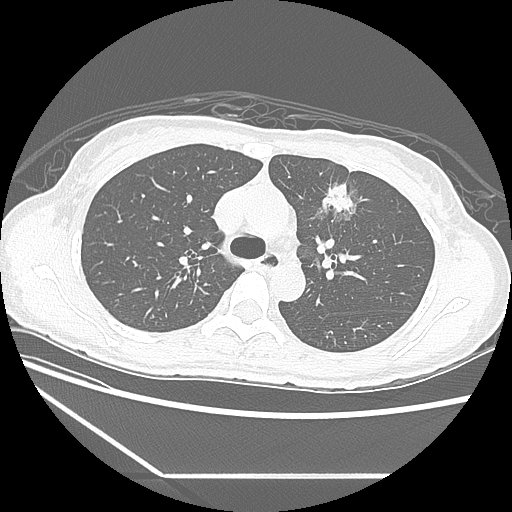

Supplement: S1 File — (ZIP) [file pone.0192002.s001.zip › Malignant/HR1.jpg]

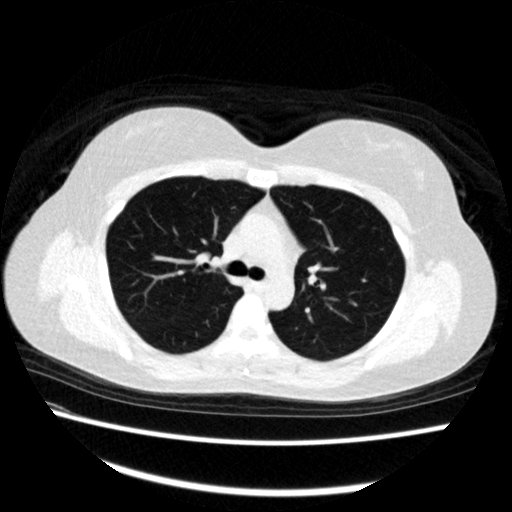

Supplement: S1 File — (ZIP) [file pone.0192002.s001.zip › Malignant/HR10.jpg]

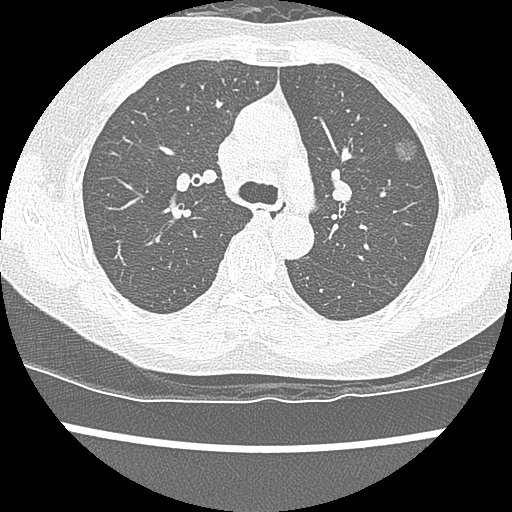

Supplement: S1 File — (ZIP) [file pone.0192002.s001.zip › Malignant/HR17.jpg]

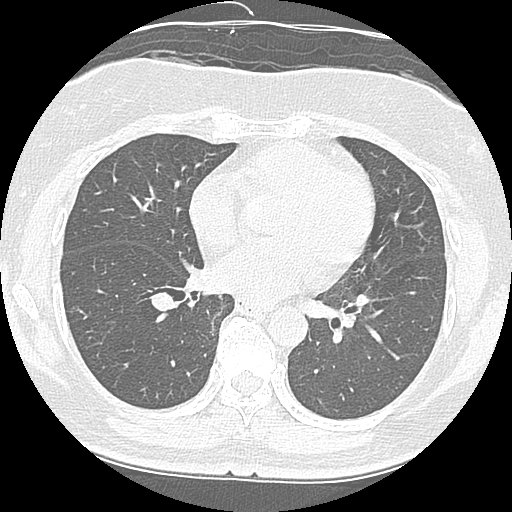

Supplement: S1 File — (ZIP) [file pone.0192002.s001.zip › Malignant/HR18.jpg]

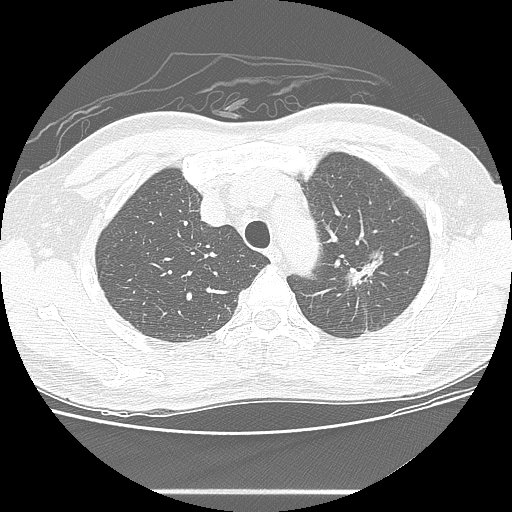

Supplement: S1 File — (ZIP) [file pone.0192002.s001.zip › Malignant/HR24.jpg]

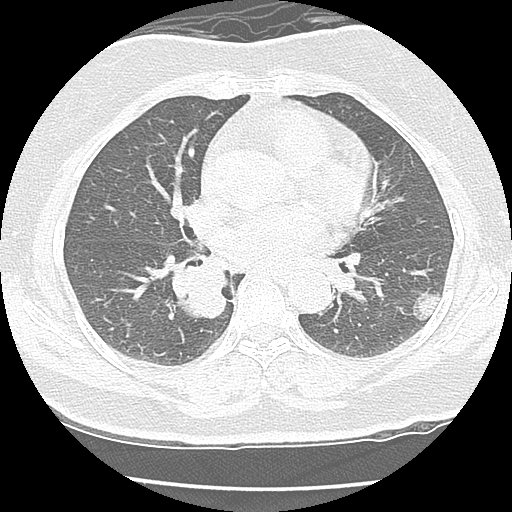

Supplement: S1 File — (ZIP) [file pone.0192002.s001.zip › Malignant/HR25.jpg]

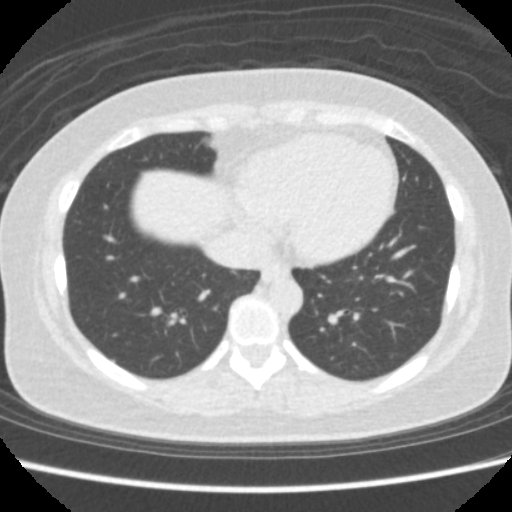

Supplement: S1 File — (ZIP) [file pone.0192002.s001.zip › Malignant/HR27.jpg]

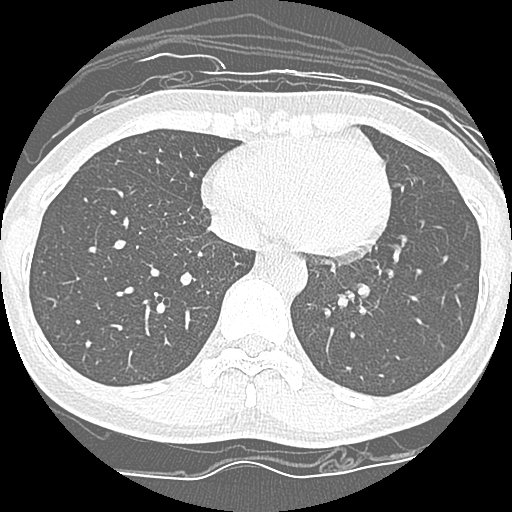

Supplement: S1 File — (ZIP) [file pone.0192002.s001.zip › Malignant/HR28.jpg]

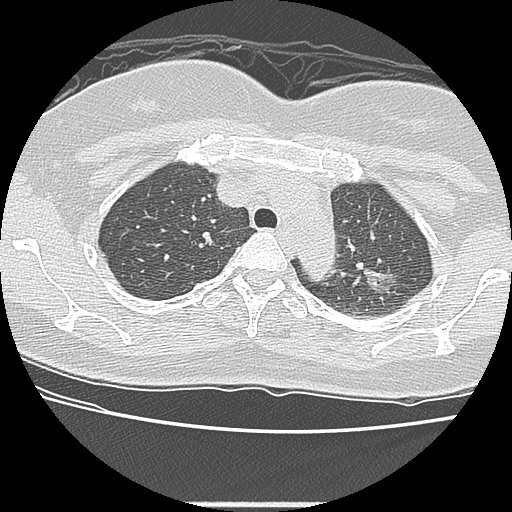

Supplement: S1 File — (ZIP) [file pone.0192002.s001.zip › Malignant/HR29.jpg]

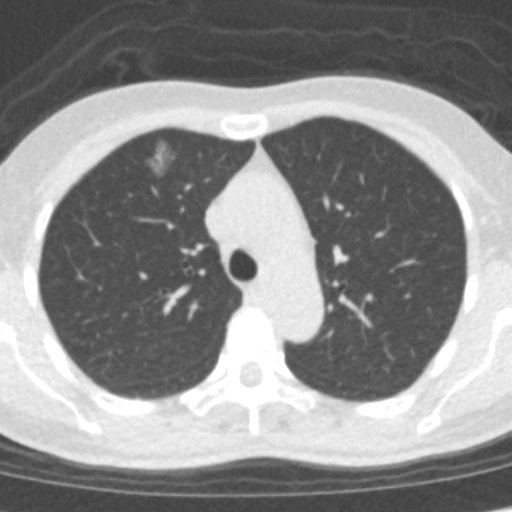

Supplement: S1 File — (ZIP) [file pone.0192002.s001.zip › Malignant/HR33.jpg]

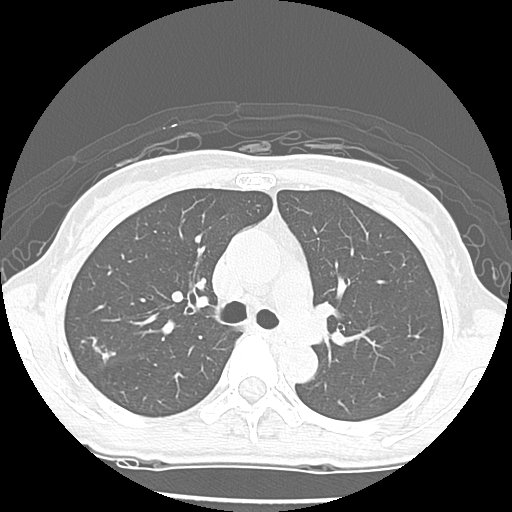

Supplement: S1 File — (ZIP) [file pone.0192002.s001.zip › Malignant/HR34.jpg]

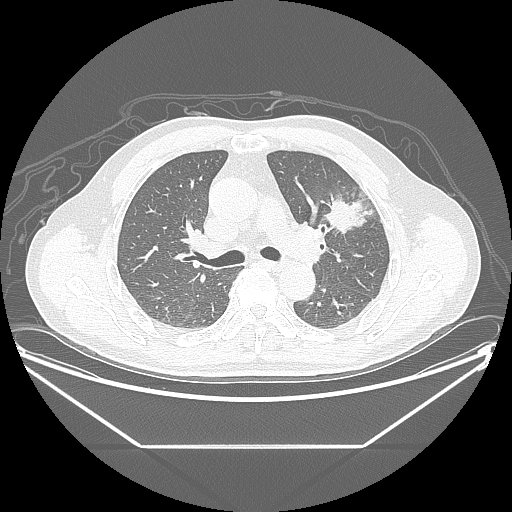

Supplement: S1 File — (ZIP) [file pone.0192002.s001.zip › Malignant/HR36.jpg]

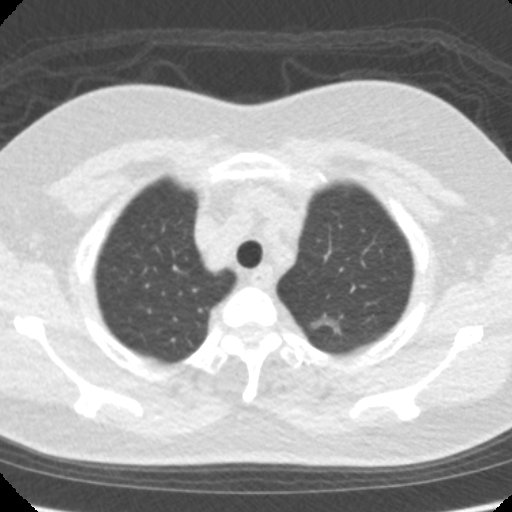

Supplement: S1 File — (ZIP) [file pone.0192002.s001.zip › Malignant/HR37.jpg]

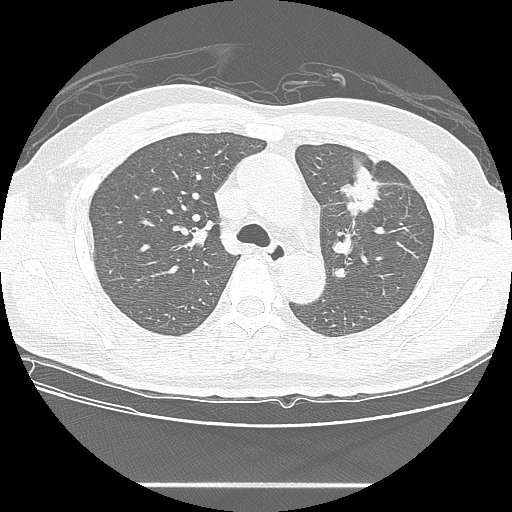

Supplement: S1 File — (ZIP) [file pone.0192002.s001.zip › Malignant/HR4.jpg]

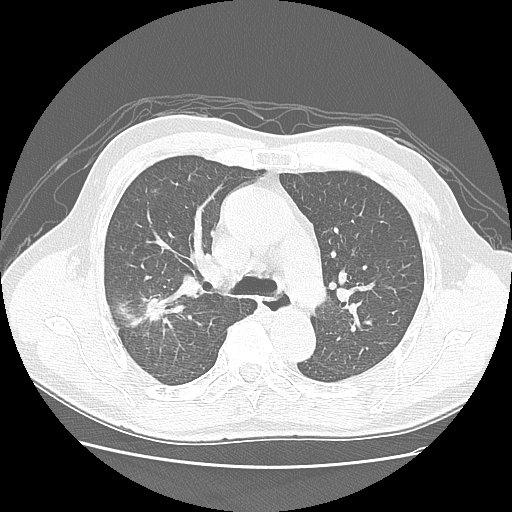

Supplement: S1 File — (ZIP) [file pone.0192002.s001.zip › Malignant/HR40.jpg]

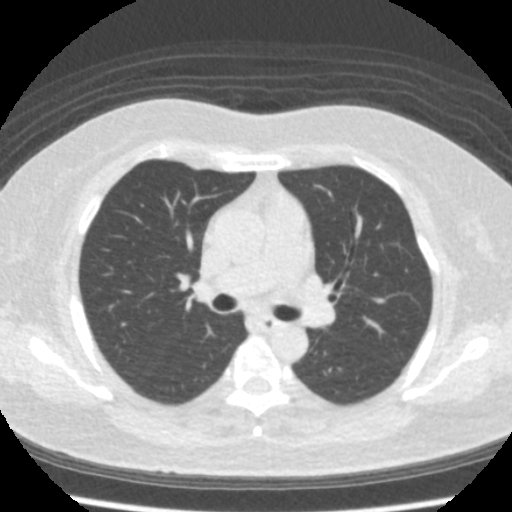

Supplement: S1 File — (ZIP) [file pone.0192002.s001.zip › Malignant/HR42.jpg]

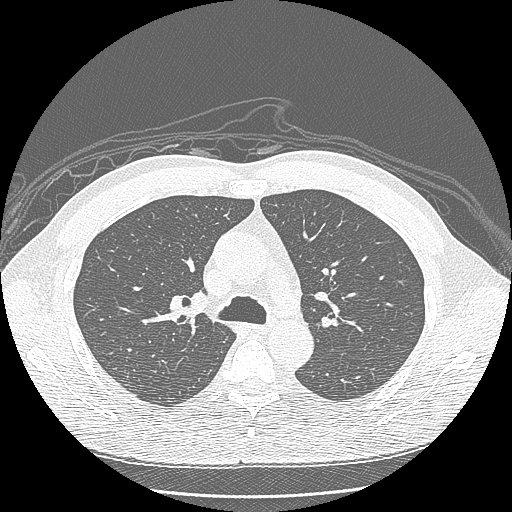

Supplement: S1 File — (ZIP) [file pone.0192002.s001.zip › Malignant/HR43.jpg]

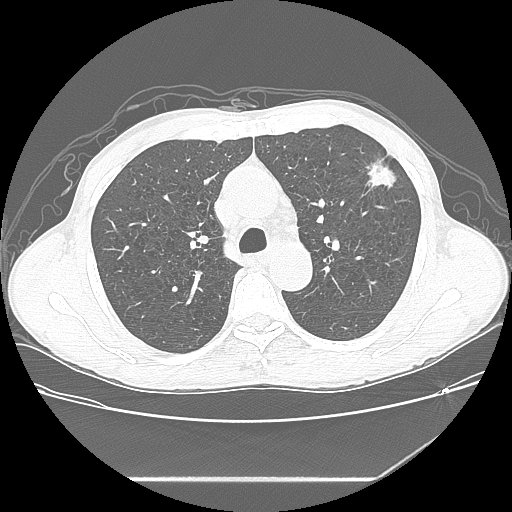

Supplement: S1 File — (ZIP) [file pone.0192002.s001.zip › Malignant/HR44.jpg]

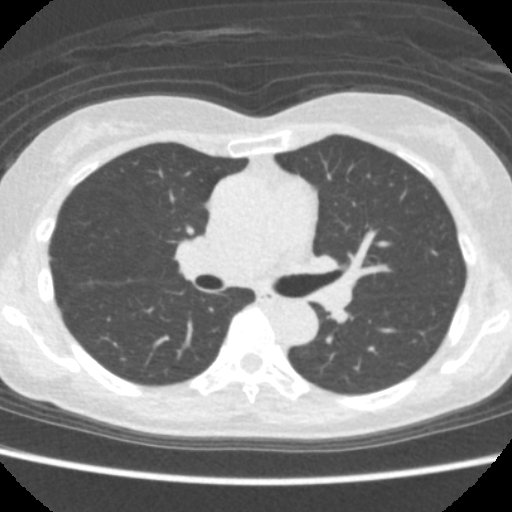

Supplement: S1 File — (ZIP) [file pone.0192002.s001.zip › Malignant/HR46.jpg]

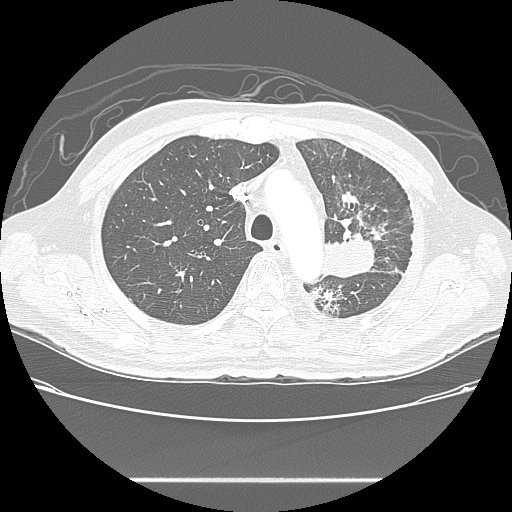

Supplement: S1 File — (ZIP) [file pone.0192002.s001.zip › Malignant/HR47.jpg]

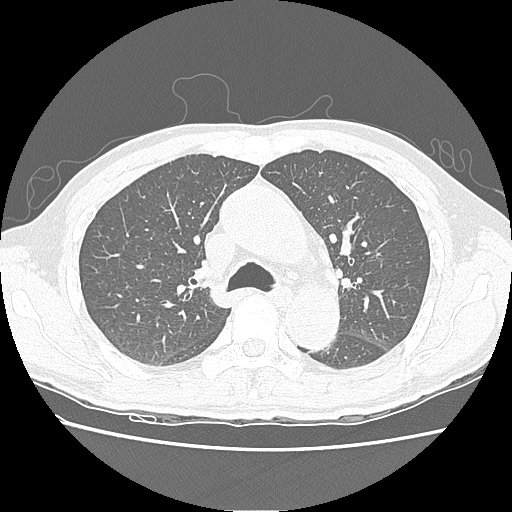

Supplement: S1 File — (ZIP) [file pone.0192002.s001.zip › Malignant/HR48.jpg]

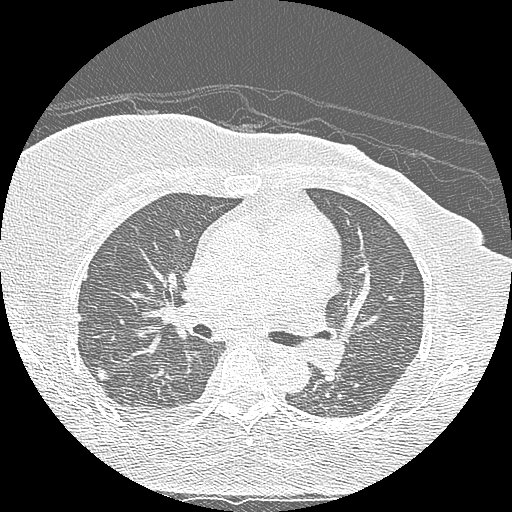

Supplement: S1 File — (ZIP) [file pone.0192002.s001.zip › Malignant/HR49.jpg]

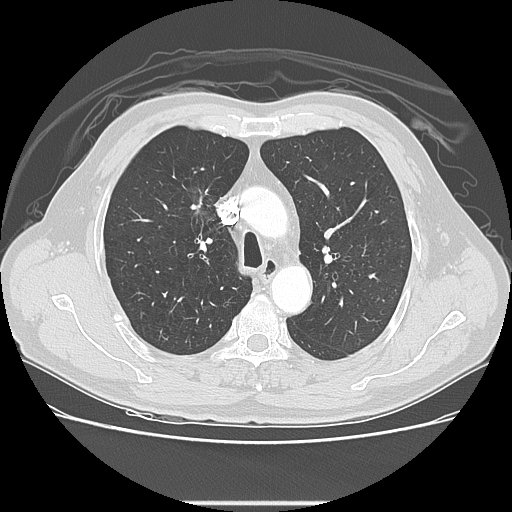

Supplement: S1 File — (ZIP) [file pone.0192002.s001.zip › Malignant/HR5.jpg]

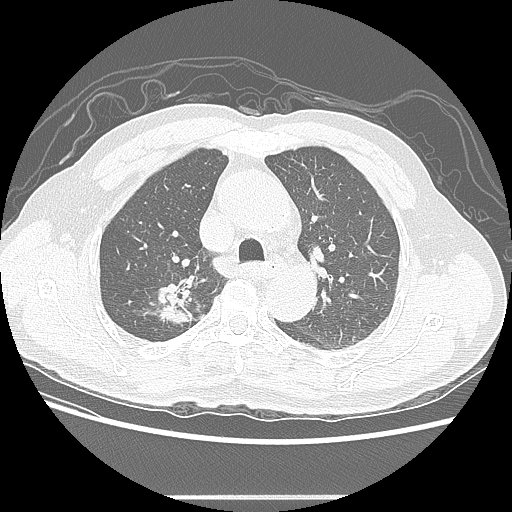

Supplement: S1 File — (ZIP) [file pone.0192002.s001.zip › Malignant/HR51.jpg]

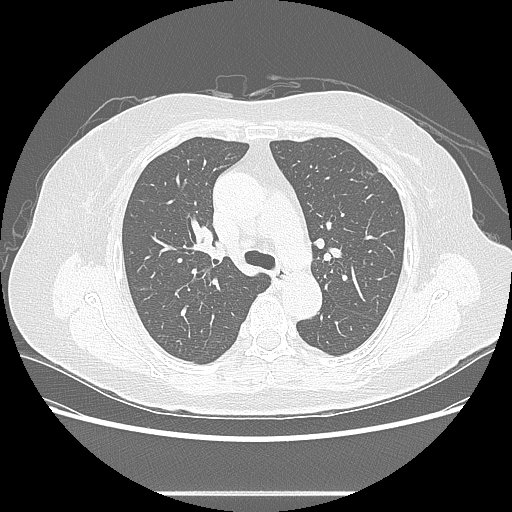

Supplement: S1 File — (ZIP) [file pone.0192002.s001.zip › Malignant/HR52.jpg]

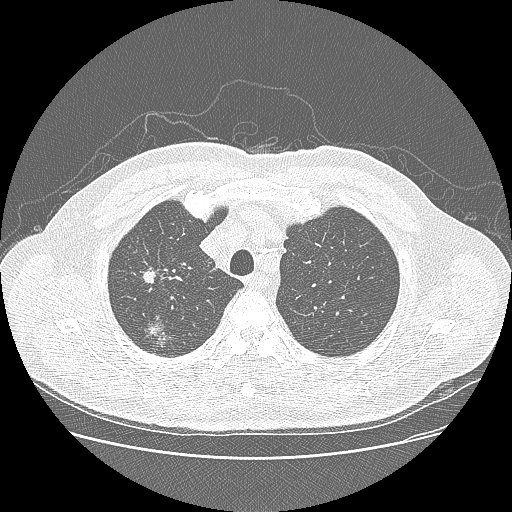

Supplement: S1 File — (ZIP) [file pone.0192002.s001.zip › Malignant/HR54.jpg]

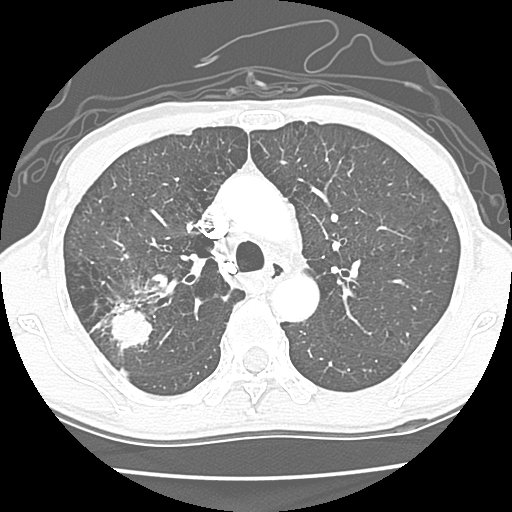

Supplement: S1 File — (ZIP) [file pone.0192002.s001.zip › Malignant/HR55.jpg]

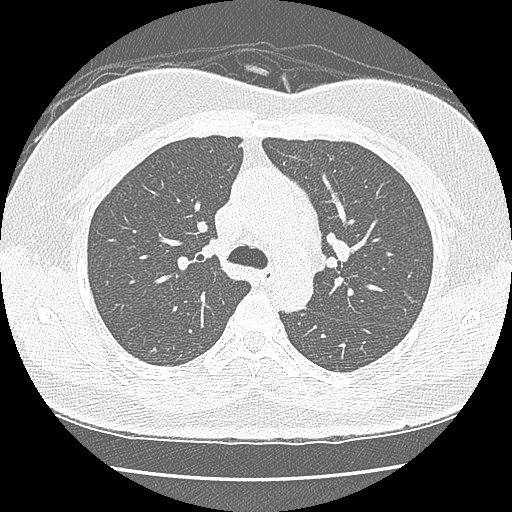

Supplement: S1 File — (ZIP) [file pone.0192002.s001.zip › Malignant/HR56.jpg]

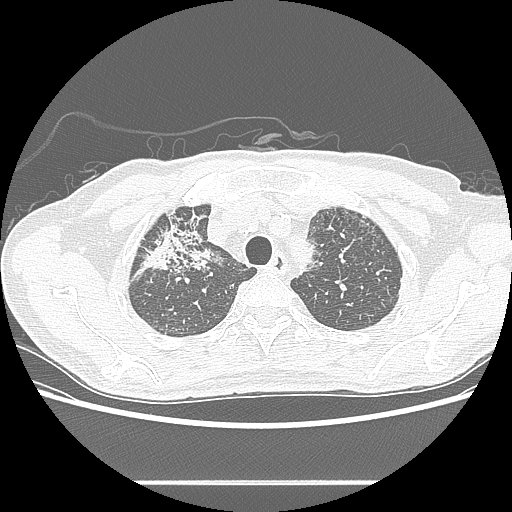

Supplement: S1 File — (ZIP) [file pone.0192002.s001.zip › Malignant/HR58.jpg]

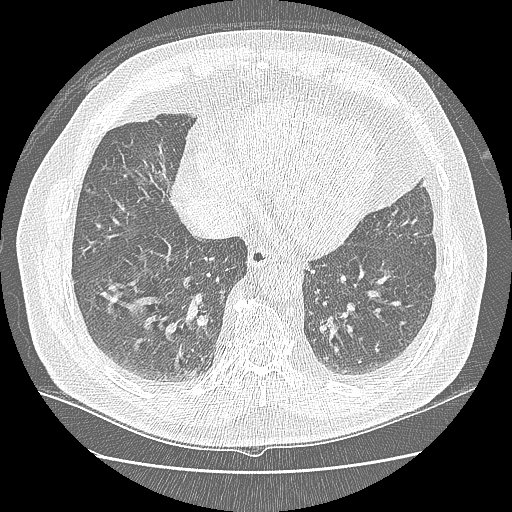

Supplement: S1 File — (ZIP) [file pone.0192002.s001.zip › Malignant/HR59.jpg]

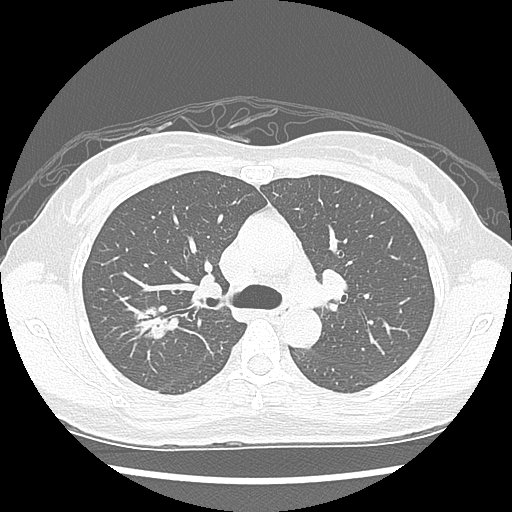

Supplement: S1 File — (ZIP) [file pone.0192002.s001.zip › Malignant/HR6.jpg]

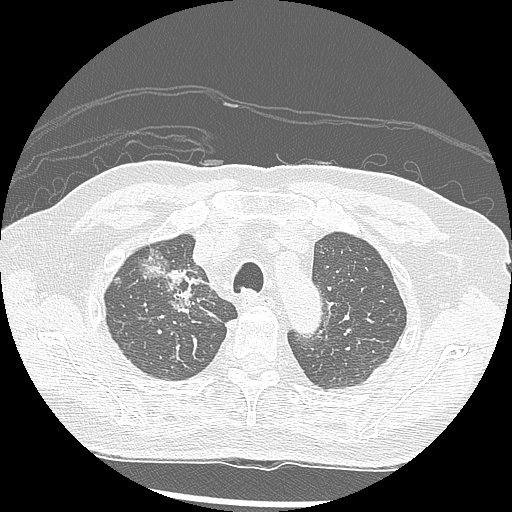

Supplement: S1 File — (ZIP) [file pone.0192002.s001.zip › Malignant/HR60.jpg]

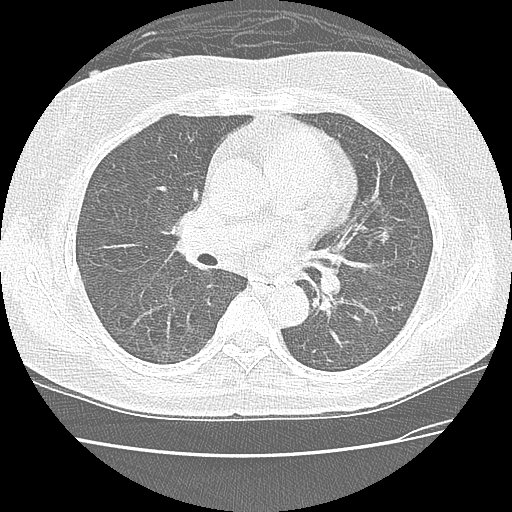

Supplement: S1 File — (ZIP) [file pone.0192002.s001.zip › Malignant/HR61.jpg]

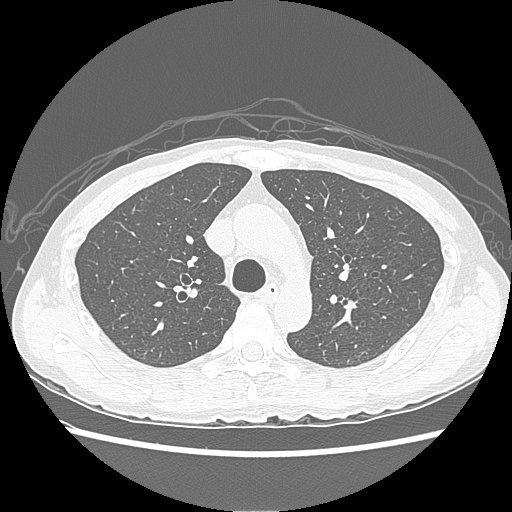

Supplement: S1 File — (ZIP) [file pone.0192002.s001.zip › Malignant/HR62.jpg]

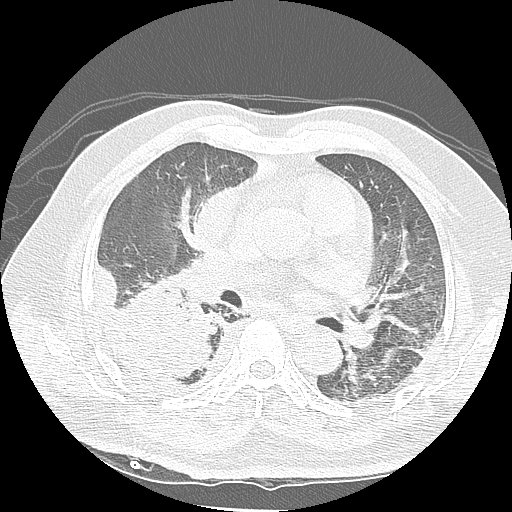

Supplement: S1 File — (ZIP) [file pone.0192002.s001.zip › Malignant/HR65.jpg]

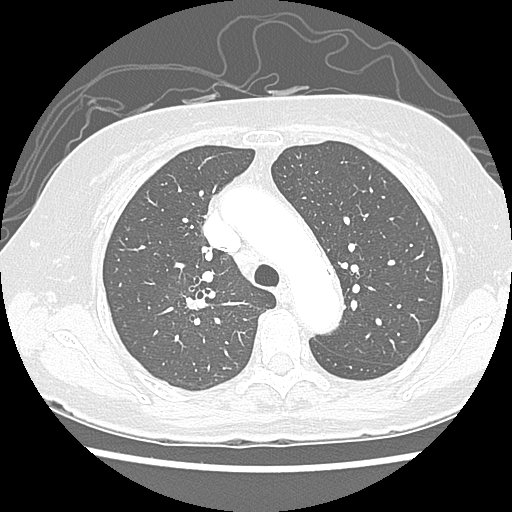

Supplement: S1 File — (ZIP) [file pone.0192002.s001.zip › Malignant/HR66.jpg]

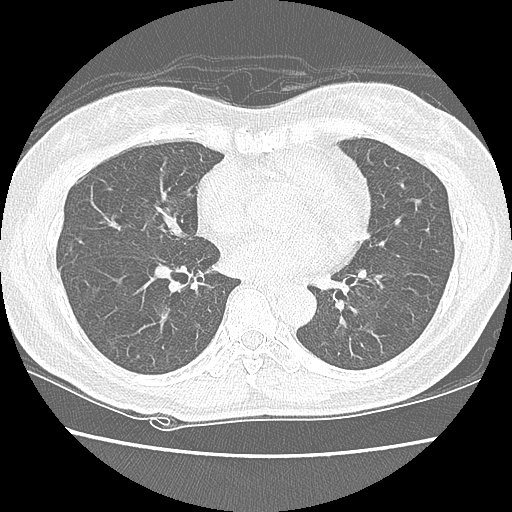

Supplement: S1 File — (ZIP) [file pone.0192002.s001.zip › Malignant/HR7.jpg]

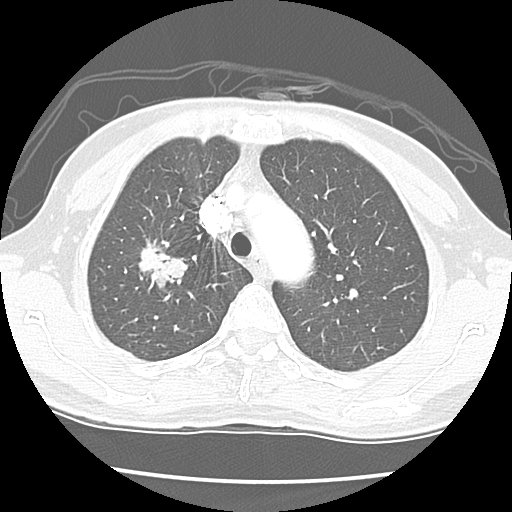

Supplement: S1 File — (ZIP) [file pone.0192002.s001.zip › Malignant/HR70.jpg]

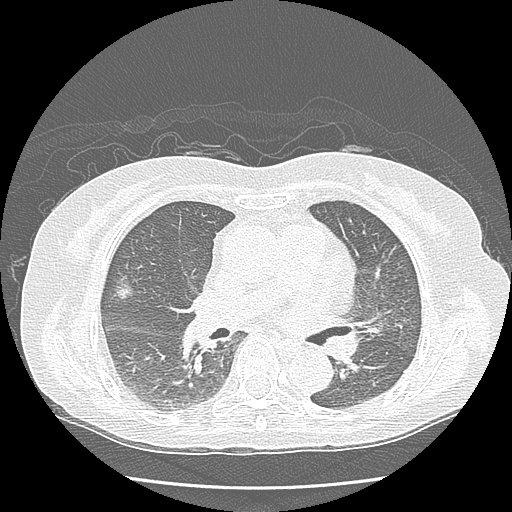

Supplement: S1 File — (ZIP) [file pone.0192002.s001.zip › Malignant/HR8.jpg]

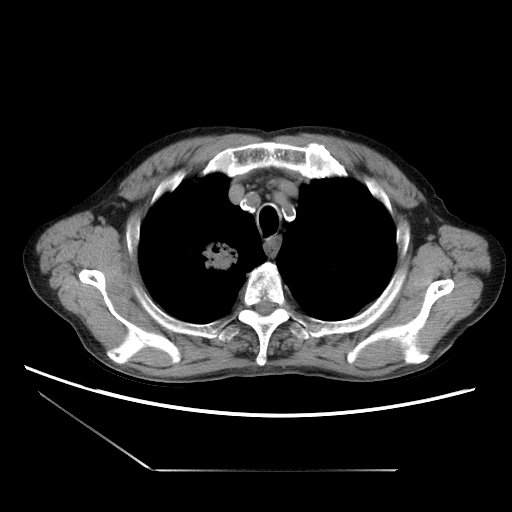

Supplement: S1 File — (ZIP) [file pone.0192002.s001.zip › Malignant/HR9.jpg]
